# Observation Safely Reduces the Use of the Computerized Tomography in Medium-to-Low-Risk Patients with Suspected Acute Appendicitis: Results of a Randomized Controlled Trial

**DOI:** 10.3390/jcm13123363

**Published:** 2024-06-07

**Authors:** Raminta Luksaite-Lukste, Igne Gecaite, Kristina Marcinkeviciute, Eimantas Dumskis, Arturas Samuilis, Tadas Zvirblis, Eugenijus Jasiunas, Augustinas Bausys, Mantas Drungilas, Martynas Luksta, Marius Kryzauskas, Marius Petrulionis, Augustas Beisa, Simonas Uselis, Gintare Valeikaite-Taugininene, Rokas Rackauskas, Kestutis Strupas, Tomas Poskus

**Affiliations:** 1 Clinic of Gastroenterology, Nephrourology and Surgery, Institute of Clinical Medicine, Faculty of Medicine, Vilnius University, LT-03101 Vilnius, Lithuania; martynas.luksta@santa.lt (M.L.); marius.kryzauskas@santa.lt (M.K.); marius.petrulionis@santa.lt (M.P.); augustas.beisa@santa.lt (A.B.); simonas.uselis@santa.lt (S.U.); gintare.valeikaite@santa.lt (G.V.-T.); rokas.rackauskas@santa.lt (R.R.); kestutis.strupas@santa.lt (K.S.); tomas.poskus@santa.lt (T.P.); 2 Department of Radiology, Nuclear Medicine and Medical Physics, Institute of Biomedical Sciences, Faculty of Medicine, Vilnius University, LT-03101 Vilnius, Lithuania; igne.gecaite@santa.lt (I.G.); eimantas.dumskis@santa.lt (E.D.); arturas.samuilis@santa.lt (A.S.); 3 Faculty of Medicine, Vilnius University, LT-03101 Vilnius, Lithuania; kristinamarcinkeviciute99@gmail.com (K.M.); tadas.zvirblis@mf.vu.lt (T.Z.); abpelikanas@gmail.com (A.B.); 4 Department of Mechanical and Material Engineering, Vilnius Gediminas Technical University, LT-03224 Vilnius, Lithuania; 5 Vilnius University Hospital Santaros Klinikos, LT-08406 Vilnius, Lithuania; eugenijus.jasiunas@santa.lt (E.J.); mantas.drungilas@santa.lt (M.D.)

**Keywords:** abdomen, acute appendicitis, abdominal ultrasound, ionizing radiation reduction, observation

## Abstract

**Objectives**—The objective was to compare the effectiveness of observation in standard-of-care computed tomography (CT) in adult patients with suspected acute appendicitis (AA). **Methods**—Patients with clinically suspected AA and inconclusive diagnosis after primary clinical examination, laboratory examination, and transabdominal ultrasound (TUS) were eligible for the study, and they were randomized (1:1) to parallel groups: observation-group patients were observed for 8–12 h and then, repeated clinical and laboratory examinations and TUS were performed; CT group (control group) patients underwent abdominopelvic CT scan. The study utilized Statistical Analysis System 9.2 for data analysis, including tests, logistic regression, ROC analysis, and significance evaluation. Patients were enrolled in the study at Vilnius University Hospital Santaros Klinikos in Lithuania between December 2018 and June 2021. **Results**—A total of 160 patients (59 men, 101 women), with a mean age of 33.7 ± 14.71, were included, with 80 patients in each group. Observation resulted in a reduced likelihood of a CT scan compared with the CT group (36.3% vs. 100% *p* < 0.05). One diagnostic laparoscopy was performed in the observation group; there were no cases of negative appendectomy (NA) in the CT group. Both conditional CT and observation pathways resulted in high sensitivity and specificity (97.7% and 94.6% vs. 96.7% and 95.8%). **Conclusions**—Observation including the repeated evaluation of laboratory results and TUS significantly reduces the number of CT scans without increasing NA numbers or the number of complicated cases.

## 1. Introduction

Acute appendicitis (AA) is the most common general surgical emergency worldwide, but its diagnosis remains challenging [1]. Traditional diagnostic tools—laboratory and clinical parameters and transabdominal ultrasound (TUS)—lack sensitivity and specificity for accurate diagnosis [2,3,4,5,6,7,8,9,10]. On the one hand, undiagnosed AA can cause lethal consequences. On the other hand, overdiagnosis leads to overtreatment in terms of negative appendectomy (NA). In cases where the majority of diagnoses are based solely on clinical and laboratory data, up to 30% of appendectomies are unnecessary, and they are associated with the risk of postoperative complications, unnecessary hospitalization, and treatment-related costs [11,12,13,14]. Several scoring systems have been proposed for improving diagnostic accuracy. However, they are more effective in excluding the diagnosis of appendicitis rather than confirming it [15,16,17].

Additional imaging has made a significant improvement in diagnostic accuracy. Traditionally, the first-line modality for AA is the transabdominal ultrasound (TUS). The pooled sensitivity and specificity are reported to be 83.1% (CI 70.3–91.1) and 90.9% (CI 59.3–98.6) in adult patients [10]. Recent European and global guidelines have proposed computed tomography (CT) for challenging cases to improve diagnostic accuracy and avoid unnecessary surgeries [16,18]. However, CT emits ionizing radiation, which poses an increased risk of cancer, especially in young patients [19]. According to our center experience, more than 50% of CT scans for suspected AA after inconclusive TUS do not show acute pathology [20]. In parallel to NA, negative CT rates should be reduced. Observation and repetitive clinical, laboratory, and TUS testing are among the alternatives for CT scan. This practice originally has been used and reported mainly in pediatric patients and pregnant women with suspected AA [20]. However, there is no strong evidence for such a practice in adults.

The aim of this study was to compare the effectiveness of observation in adult patients with suspected AA to standard-of-care conditional CT strategy diagnostics when primary TUS is inconclusive. Moreover, it aimed to identify possible changes in inflammatory markers or their combinations that would be predictive for AA in the observation group.

## 2. Materials and Methods

### 2.1. Study Design

The study was conducted in accordance with the Declaration of Helsinki, approved by Vilnius Region Bioethics Committee (approval No: 2019/3-1107-610, date: 18 March 2019) and registered in Clinicaltrials.gov database (NCT04117061); patient consent was given by all patients. A randomized, controlled, open-label, (1:1) parallel-group trial was performed. Patients were recruited between December 2018 and June 2021 at Vilnius University Hospital Santaros Klinikos in Lithuania.

### 2.2. Participants

The adult patients admitted to the emergency department for suspected AA were eligible if AA was not reliably confirmed or ruled out after initial clinical, laboratory, and TUS evaluation (not visualized appendix by TUS as the primary criterion, visualized appendix with possible inflammation, or visualized but normal appendix by TUS in the presence of strong clinical suspicion and changes in laboratory tests) and no other acute pathology was confirmed. These patients were considered to be in medium-to-low-risk patients for AA and were included in study. Exclusion criteria affected patients with symptom duration >48 h, patients with clear diagnosis after a primary clinical evaluation, patients having high C-reactive protein level (>100 mg/L) or signs of peritonitis, and pregnant women.

### 2.3. Sample Size Calculation

The sample size for the present trial was calculated based on the hypothesis of the reduction in CT scan rate from 100 to 83% using a two-tailed Fisher’s exact test. To achieve 99% power with a two-sided *p* < 0.050, a group size of 73 patients was required. Assuming a dropout rate of 10%, the total sample size was calculated at 160 patients with 80 in each of the two groups.

### 2.4. Randomization

Participants were randomly assigned to immediate CT or observation group after informed consent with 1:1 allocation ratio using consecutively numbered, sealed opaque envelopes. The randomization list was created using computer-based block-type randomization (block size of 4).

### 2.5. Diagnostic Workup and Treatment in CT and Observation Groups

The patients in the CT group were sent directly for abdominopelvic CT scans. After the CT, each patient was either operated on or discharged home, or another treatment was administered when an alternative diagnosis was identified, based on the CT scan results.

**Figure 1:**
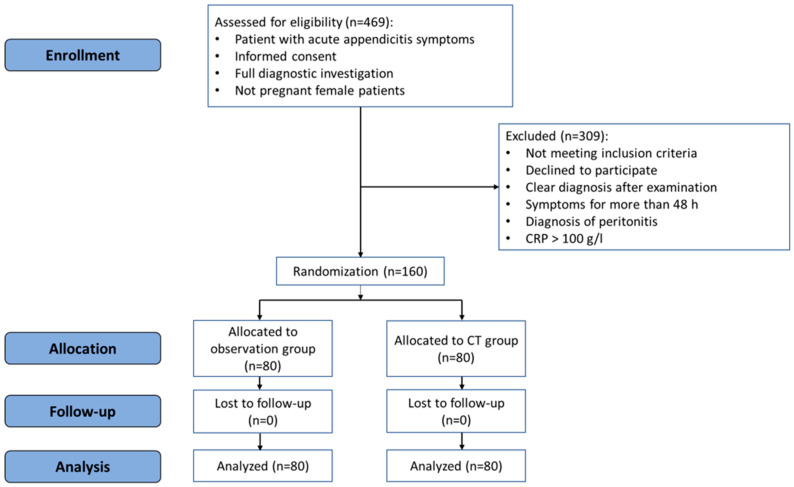
CONSORT flowchart of the randomized controlled trial.

The patients in the observation group were observed for 8–12 h at the Emergency Department. After the observation period, a clinical re-evaluation was performed by the surgeon, and laboratory tests and TUS examination were repeated. If repeated TUS was conclusive (confirming or excluding acute appendicitis), the patient underwent surgery or was discharged home, or another treatment was administered if an alternative diagnosis was confirmed. If repeated TUS was inconclusive, a CT scan was then performed, and the final decision was made according to the results of the CT scan. The flowchart of the study is presented in Figure 1.

### 2.6. Clinical and Laboratory Tests

For every patient, white blood cell (WBC), neutrophil (NEU), lymphocyte (LYMP), and monocyte (MON) counts and percentage values and C-reactive protein (CRP) levels were measured. These laboratory tests were repeated after 8–12 h in observation-group patients. The changes in laboratory markers over the time observation were called delta and were counted using the following formula: secondary value minus primary value = delta value. Every patient had Alvarado and AIR scores counted at primary evaluation by treating surgeon.

### 2.7. Imaging Procedures

TUS was performed in the emergency department by board-certified radiologists or radiology residents (with at least two years’ experience). The ultrasound was performed with a convex (2–5 MHz) probe followed by a more detailed examination of the right iliac fossa with a high-frequency linear probe using the graded compression technique. Four different ultrasound machines were used during the study period—Toshiba Aplio 500, GE Logiq S8, GE Logiq 9, and Toshiba Xario 400. If the appendix was visualized on TUS, it was classified into the following groups:

- Normal appendix—diameter of the appendix ≤6 mm, wall thickness of the appendix <2 mm, compressible appendix without secondary findings of free fluid in the right iliac fossa, lymphadenopathy, and infiltration of surrounding tissue.
- AA—diameter of appendix >6 mm, wall thickness of the appendix ≥2 mm, uncompressible appendix with or without secondary findings of free fluid in the right iliac fossa, lymphadenopathy, and infiltration of surrounding tissue.
- Probable AA—diameter of appendix 6–7 mm (or less), wall thickness of the appendix ~2 mm (or less), compressible/partially compressible appendix with or without secondary findings of free fluid in the right iliac fossa, lymphadenopathy, and infiltration of surrounding tissue.

CT scans were performed using GE Discovery 750 HD (General Electric Healthcare, Waukesha, WI, United States) with intravenous contrast enhancement (iohexol, Omnipaque 350 mg/mL, GE Healthcare, Oslo, Norway; bolus 1.5 mL/kg body weight at 3 mL/s flow rate) in portal venous phase. CT parameters were as follows: tube voltage—120 kV, automatic tube current modulation—SmartmA/AutomA, FOV—Large Body, detector coverage—40 mm, slice thickness—2.5 mm, pitch—1.375 mm, and gantry rotation time—0.5 s. All images were then reconstructed with slice thickness of 1.25 mm using standard algorithm. Scans were assessed by board-certified radiologists specializing in emergency radiology. Following cut-off values were used for AA diagnosis: diameter of appendix >6 mm and wall thickness of the appendix ≥2 mm, with possible secondary signs—free fluid in the right iliac fossa, lymphadenopathy, and fat stranding.

Patients were operated on in cases when radiological evidence of AA was present. The appendices that appeared normal during the operation were not removed.

### 2.8. Data Collection

Patient data were entered into the prospectively maintained database, including the following: age, sex, radiological diagnosis (ultrasound and/or CT findings), treatment strategies, and operative and histological findings. A final diagnosis was assigned to every patient by an expert panel, based on histopathology, imaging, and surgical findings; clinical information; and at least 6 months of follow-up.

### 2.9. Outcomes

The primary outcome of the study was the proportion of patients requiring CT for final diagnosis in the observation group. The secondary outcomes were rate of NA and complicated AA in observation and CT scan groups and the impact of repeated laboratory tests and TUS results over time to confirm the final diagnosis. After the trial commenced, there were no changes to trial outcomes.

### 2.10. Statistical Analysis

Descriptive statistics such as frequency tables and the mean with standard deviation (SD) were used to describe quantitative and qualitative data, respectively. The normality of quantitative variables was assessed using the Kolmogorov–Smirnov test. Differences between two independent quantitative and qualitative groups were evaluated using Student’s t-test and chi-squared test, respectively. Generalized McNemar test was used to compare repeated quantitative tests with multiple categories. Univariate binary logistic regression models were concluded for the prediction of AA. Prediction accuracy was measured by using the classification table method and by estimating the area under the receiver operating characteristic (ROC) curves (AUCs). The determination of cut-off values was based on maximum Youden’s index. DeLong method was used to compare the ROC curves. A two-tailed *p*-value less than 0.05 was considered to be significant. Statistical analysis was performed using Statistical Analysis System (SAS) package version 9.2.

## 3. Results

The study included 160 patients who were divided into a CT group and observation group between December 2018 and June 2021. There were no dropouts from the study. Figure 1 represents the flowchart of the study.

The average time from the start of symptoms until hospital admission was 17.7 (SD 13.14) in the CT group and 13.4 (SD 12.15) hours in the observation group (*p* = 0.035). Detailed demographic characteristics of the patients are described in Table 1.

TUS was performed for all the patients in both groups. There were two cases where the TUS result was AA, but clinically symptoms were equivocal. Therefore, a further investigation underwent a CT scan. AA or suspected AA was observed in forty-four (55.0%) cases, another acute pathology was found in six (7.5%) cases, and the rest (37.5%) had uninflamed appendices. Patients who were not diagnosed with AA after the observation were discharged home from the emergency department and had no readmissions or complications during the 6-month follow-up period.

A CT scan was performed in 29 (36.3%) patients in the observation group. Eleven (13.8%) patients with AA or suspected AA were observed. Overall, there were two false-positive observations and one false-negative observation in both groups.

The results of repeated laboratory tests in the observation group are presented in Table 2 and the results of the repeated TUS examinations of the observation group are presented in Table 3.

**Figure 2:**
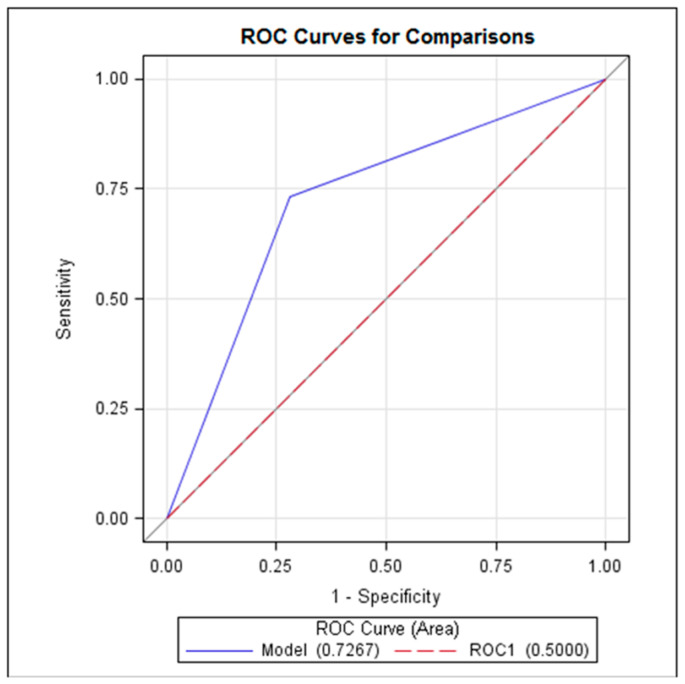
ROC curve representing the delta CRP performance in diagnostics of AA.

Logistic regression analysis showed that only the delta CRP value showed a statistically significant correlation with the diagnosis of AA (*p* < 0.05). The estimated cut-off value of delta CRP was 9.4 mg/L with a sensitivity of 73.3% (CI 57.5–89.2), specificity of 72% (CI 59.6–83.8), positive predictive value of 61.1% (CI 45.2–77.0), and negative predictive value of 81.8% (70.4–93.2). The ROC curve demonstrated that delta CRP reached AUC 0.7267 (*p*< 0.05) (Figure 2).

Overall, 76 patients underwent surgical treatment: 44 (55.0%) patients in the CT group and 32 (40.0%) patients in the observation group. All the patients had laparoscopic surgeries. There was one case described as gangrenous appendicitis that was reported as a low-grade mucinous neoplasm after pathology examination. There were no cases of NA in the CT group and one diagnostic laparoscopy in the observation group, which resulted in an overall NA rate of 3.1%. No patients were treated conservatively in both groups.

**Figure 3:**
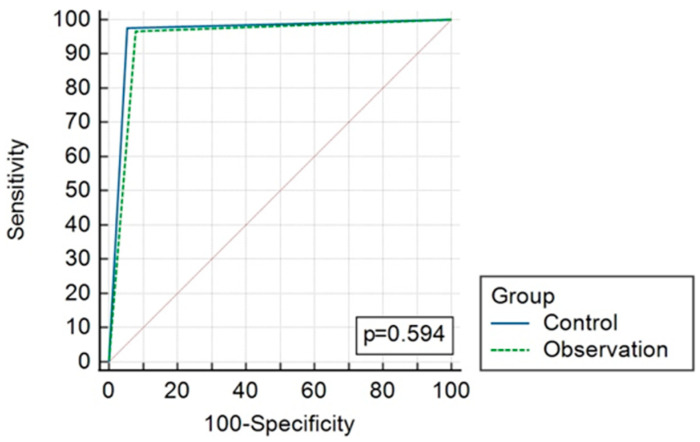
ROC curves comparing results of CT (control) and observation group diagnostic strategies.

The overall diagnostic accuracy of both groups is presented in Table 4 and Figure 2 and Figure 3. AUC reached 0.96 (CI 0.893–0.992) and 0.94 (CI 0.868–0.983), *p* = 0.59, in the CT and observation groups, respectively.

## 4. Discussion

Our study found that observation with repeated TUS and laboratory tests can reduce the rate of CT scan use up to 63.7% without increasing complicated AA and NA rates in patients with inconclusive primary TUS. We also found that an increased CRP value by 9.4 mg/L is predictive of AA after 8–12 h of observation.

A similar study by Andersson et al. used AIR diagnostic score evaluation and successfully decreased the CT use by 44% [21]. A recently performed prospective randomized study presented a sensitivity of 100% for AIR scores since there were no false-negative AA diagnoses, implying that clinical scores can minimize the number of imaging tests and waiting time for AA diagnosis, while also improving diagnostic accuracy [22]. Therefore, all patients in our study were evaluated with Alvarado and AIR scores that helped us to detect patients with probable AA. It seems that repeated ultrasound brings additional benefits in avoiding ionizing radiation when conducting an imaging test is necessary for the diagnosis of AA. The conditional use of CT after inconclusive TUS seems to be a promising tool to reduce the use of CT by 50–70% in comparison with direct CT application [20,23]. Moreover, it is applicable for obese people when ultrasound is uninformative due to predominant subcutaneous fat [24]. However, it is still not effective enough as, in 53.8% of cases, when CT was used after inconclusive TUS, it did not reveal any urgent pathology [20], even though CT imaging was used in only 25% of patients with suspected AA. The present study showed a similarly low rate of normal CT scans in the CT and observation groups (37.5% vs. 34.5%, accordingly). If the diagnosis remains unclear after repeated ultrasound examinations (by two independent radiologists) and a CT scan is indicated, some authors suggest that low-dose CT may be an option since diagnostic accuracy and clinical results for suspected appendicitis did not differ between the 2-mSv CT and conventional-dose CT (CDCT) groups [25]. The OPTICAP study showed that the ability not only to diagnose acute appendicitis but also to differentiate between complicated and uncomplicated cases for individuals with a high risk of acute appendicitis was equally accurate with contrast-enhanced low-dose CT and normal CT [26]. This may be applicable when we need to confirm a case of uncomplicated appendicitis for conservative treatment with antibiotics as this treatment in the APPAC study was proved to be safe only for uncomplicated cases [27]. However, radiologists need to be trained to analyze these low-dose CT images since these are not routinely used. The first study analyzing the role of observation in the pediatric population was presented in 1975, showing a reduction in the NA rate from 15% to 1.2% [28]. However, since then, all the studies have been in the pediatric population. Recently, Anderson et al. reported on the randomized trial comparing direct imaging to observation, clinical re-evaluation, and selective imaging, which showed better outcomes in the observation arm [21]. In our study, we performed repeated laboratory tests and analyzed the dynamics of test values over the time period of 8–12 h in the observation-group patients. The only marker that significantly correlated with the diagnosis of AA was the CRP value. Our estimated delta CRP cut-off value of 9.4 mg/L reached a sensitivity of 73.3% and specificity of 72% (CI 59.6–83.8) in differentiating AA. A previous study in the child population demonstrated that children with a CRP level on admission ≥10 mg/L were over seven times more likely to have appendicitis than those with CRP level <10 mg/L [29]. Suggestions were made that repeated TUS may also show better diagnostic performance compared to initial TUS as the progression of the inflammatory process in the appendix would make it easier to detect [10]. There were few earlier studies analyzing the value of repeated ultrasound, with all of them showing positive outcomes [30,31]. In our study, repeated TUS led to significantly lower rates of CT scans and revealed a final diagnosis for close to 49% of cases. Therefore, low-risk patients could be observed until the following day in order to repeat the ultrasound scan and avoid ionizing radiation. The main indicator of the successful diagnostic workflow of AA is a low NA rate. NA is associated with excess mortality that is almost at the same level as among patients with perforated appendicitis [32]. Also, NA is significantly associated with an increased risk of ectopic pregnancy [33]. Currently, a NA rate of 10% has been considered to be acceptable in young healthy males and 20% in women of reproductive age because of the other pelvic inflammatory conditions, which can complicate the evaluation [34]. The NA rate was 0% in the CT group and 3.1% in the observation group. Low NA rates seem to be closely related to the amount of imaging used in diagnostics. However, the optimal use of imaging in AA is still not clear, and using only the NA rate as a measure of quality is not satisfactory [35]. Some authors promote imaging only in selected cases, using scoring systems to stratify patients when imaging is not necessary due to low yield [36], whereas others have shown a decrease in NA with the use of extensive preoperative imaging [37,38]. In our previous cohort study, conditional CT strategy application resulted in a 3.4% NA rate [20]. Also, a similar positive impact was seen in the pregnant patient population while using the conditional MRI strategy [19]. Despite prolonged time to surgery in the observation group, there was no significant difference between the rates of complicated cases. It is known that a short, in-hospital surgical delay of up to 24 h is safe in uncomplicated AA and does not increase complications and/or the perforation rate in adults [39]. Moreover, a comparable study [22] found the AIR score is safe in predicting the need for surgical treatment for patients with high AIR scores without the need for imaging examination as all these patients in both groups, who initially underwent CT scanning or straightforward surgical treatment, had a histopathologically confirmed diagnosis of acute appendicitis. Based on Andersson and colleagues’ recently performed randomized control trial, 69% of patients with an intermediate AIR score could be treated without any imaging tool [21]. According to the recent guidelines, the antibiotic-first strategy can be considered safe and effective in selected patients with uncomplicated AA [16]. Moreover, with the repeated score-based surveillance of patients with early inconclusive appendicitis, fewer patients require appendicitis treatment at all, as 27% of patients in a recent randomized trial [40] who were observed for longer without treatment were discharged with a diagnosis of non-specific abdominal pain. Nevertheless, a recent meta-analysis reported a recurrence rate of symptoms within 1 year of 27.4% following antibiotic-first treatment [41]. Overall, both diagnostic pathways (CT group and observation group) resulted in high sensitivity and specificity (97.7% and 94.6% vs. 96.7% and 95.8%, *p* > 0.05).

The main drawback of the study was a single tertiary-center setting, so the applicability of the results may be limited in other environments. Moreover, the re-evaluation of diagnostic scales such as the Alvaro and AIR scales after the observation period could potentially bring additional insights into the results.

## 5. Conclusions

Observation including the repeated evaluation of laboratory results and ultrasound significantly reduces the number of performed CT scans without increasing the NA rate or the number of complicated cases and results in the same diagnostic accuracy as the conditional CT strategy. An increased CRP value might be a potential diagnostic marker for AA in patients under observation.

## Figures and Tables

**Table 1:** Patient characteristics of the randomization groups.

Patient Characteristics		CT Group n (%)	Observation Group n (%)	*p*-Value
Overall		80 (100)	80 (100)	
Age	Overall	33.7 ± 14.71		0.041
		36.0 ± 16.12	31.3 ± 12.82	
Sex	Women	52 (65.0)	49 (61.3)	0.623
	Men	28 (35.0)	31 (38.8)	
Primary transabdominal ultrasound	Overall	80 (100)	80 (100)	
	Acute appendicitis	2 (2.5)	2 (2.5)	0.244
	Suspected acute appendicitis	8 (10.0)	17 (21.3)	
	Normal appendix	6 (7.5)	7 (8.8)	
	Non-visualized	64 (80.0)	54 (67.5)	
Computed tomography	Overall	80 (100.0)	29 (36.3)	<0.05
	Acute appendicitis	39 (48.8)	9 (31.0)	<0.05
	Suspected acute appendicitis	5 (6.3)	2 (6.89)	
	Normal appendix	30 (37.5)	10 (34.5)	
	Other disease	6 (7.5)	8 (27.9)	
Alvarado score		4.5 (2.03)	4.2 (1.81)	0.324
AIR score		4.2 (1.76)	3.9 (1.61)	0.243
WBC (×10^9^/L)		11.4 (3.96)	11.4 (4.12)	0.969
NEU (%)		9.0 (3.82)	9.0 (4.06)	0.995
LYM (%)		1.5 (0.65)	1.5 (0.73)	0.431
MON (%)		0.8 (0.35)	0.7 (0.28)	0.051
CRP (mg/L)		20.5 (26.35)	17.2 (20.64)	0.049
Diagnosis	Uncomplicated acute appendicitis	33 (41.3)	28 (35.0)	0.053
	Complicated acute appendicitis	10 (12.5)	2 (2.25)	
	Other acute pathology	7 (8.8)	9 (11.3)	
	No acute pathology	30 (37.5)	41 (51.3)	
Surgical treatment	Laparoscopic appendectomy	44 (55.0)	31 (38.8)	0.08
	Diagnostic laparoscopy	0 (0)	1 (1.2)	
	No surgery	36 (45.0)	48 (60.0)	
Surgical findings	No appendicitis	0 (0)	1 (3.1)	0.461
	Catarrhal appendicitis	0 (0)	0 (0)	
	Secondary appendicitis	0 (0)	0 (0)	
	Phlegmonous appendicitis	28 (63.6)	24 (75.0)	
	Gangrenous appendicitis	8 (18.2)	4 (12.5)	
	Gangrenous perforated appendicitis	7 (15.9)	2 (6.3)	
	Other pathology	1 (2.3)	1 (3.1)	
Histopathological findings	No appendicitis	0 (0)	0 (0)	0.457
	Catarrhal appendicitis	0 (0)	0 (0)	
	Secondary appendicitis	1 (1.3)	0 (0)	
	Phlegmonous appendicitis	26 (59.1)	23 (71.9)	
	Gangrenous appendicitis	7 (15.9)	5 (15.16)	
	Gangrenous perforated appendicitis	8 (18.2)	3 (9.37)	
	Other pathology	2 (4.5)	1 (3.1)	

**Table 2:** Comparison of laboratory marker dynamics in observation group.

	Value	Acute Appendicitis	No Acute Appendicitis	*p*-Value
Delta WBC	Median (IQR)	−2.02 (3.97)	−2.73 (2.99)	0.877
Delta NEU	Median (IQR)	−2.24 (4.45)	−2.75 (4.00)	0.935
Delta NEU (%)	Median (IQR)	−6.20 (12.10)	−10.65 (15.40)	0.267
Delta LYMP	Median (IQR)	0.10 (0.80)	0.25 (1.10)	0.413
Delta LYMP (%)	Median (IQR)	4.25 (10.40)	6.95 (12.70)	0.335
Delta MON	Median (IQR)	0.00 (0.20)	0.00 (0.30)	0.650
Delta MON (%)	Median (IQR)	1.50 (2.70)	1.60 (3.70)	0.349
Delta CRP	Median (IQR)	20.72 (34.93)	1.79 (12.48)	0.004

**Table 3:** Dynamics of repeated TUS results in the observation group. Generalized McNemar *p* < 0.001.

	Repeated TUS	Suspected AA N (%)	Non-Visualized Appendix N (%)	Normal Appendix N (%)	AA N (%)	Other Pathology N (%)	Total
Primary TUS							
Suspected AA		5 (29.4)	1 (5.9)	4 (23.5)	7 (41.2)	0	17
Non-visualized appendix		2 (3.7)	33 (61.1)	7 (13.0)	10 (18.5)	2 (3.7)	54
Normal appendix		2 (28.6)	1 (14.3)	4 (57.1)	0	0	7
AA		0	0	0	2 (100.0)	0	2
Total		9 (11.3)	35 (43.8)	14 (18.8)	19 (23.8)	2 (2.5)	80

**Table 4:** Comparison of conditional CT strategy and observation strategy statistic values.

	Conditional CT Strategy (Control Group)	Observation Strategy (Intervention Group)
Sensitivity	97.7%	96.7%
Specificity	94.6%	95.8%
NPV	97.2%	97.8%
PPV	95.5%	93.9%
Accuracy	96.3%	96.3%

## Data Availability

Data are contained within the article.
